# Bucillamine in the treatment of patients with mild to moderate COVID-19: an interview with Michael Frank

**DOI:** 10.2217/fmb-2021-0277

**Published:** 2022-01-19

**Authors:** Michael Frank

**Affiliations:** 1 Revive Therapeutics, Chairman & Chief Executive Officer, Toronto, ON M5C 1P1, Canada

**Keywords:** anti-inflammatory, bucillamine, clinical trial, COVID-19, COVID-19 therapeutics, SARS-CoV-2

This interview was conducted by Atiya Henry, Commissioning Editor of *Future Microbiology*. Michael Frank is the Chairman of the Board and CEO of Revive Therapeutics. Mr Frank has a strong background in operations, business development, M&A and the capital markets. Mr Frank is currently the President of Mifran Consulting, providing advisory services to emerging technology companies in a number of key verticals. In the past, Mr Frank has served as the CEO and Director of Sprylogics International and the Internet of Things Inc., as well as holding senior management positions at Ernst & Young, Data General and NCR. Mr Frank has had successful exits in the technology sector including one to Intuit Corporation, and has been instrumental in advising several early-stage software companies including a number in the cannabis sector over the last few years. Additionally, prior to being CEO, Mr Frank had consulted to Revive’s senior management team on various strategic initiatives.

## Could you please begin by introducing yourself, your background & tell me a bit about your work to date? What drew you to this area of work?

My background is in technology and biotechnology, but predominantly I was an investor in this company since around 2015. The company is very focused on repurposing drugs for several areas. I was an investor, and I always liked the opportunity to build up a portfolio in different areas, mainly infectious diseases. We were also doing work in the pharmacology area of the cannabis sector, and that led us to do work in psychedelics – so the company has had a good history of repurposing drugs with strong intellectual property. I came in as CEO back in December 2019 and reshaped some of the focus, based on the intellectual property that Revive had, and that took us to where we are at this point.

## Your current role is Chairman & CEO of Revive Therapeutics. What are Revive’s key missions & values?

Our mission really is to look for opportunities in areas that we can repurpose drugs and take a new product to the market. The sectors that we are addressing right now are focused on infectious diseases with our current COVID trial [1], and also, we are doing a lot of work in psychedelics with psilocybin. We have a strong framework around psilocybin, as we work with several US institutions and universities in terms of formulation and clinical development. Those are our two main pillars. We also have some good intellectual property in the cannabis sector around cannabidiol (CBD) and have US FDA orphan drug designation to treat auto immune hepatitis and ischemia with CBD.

## Revive is currently conducting a Phase III trial which involves the study of bucillamine in the treatment of patients with COVID-19. Could you tell me a bit about bucillamine & the rationale behind why it has been chosen for study?

Bucillamine is a drug that is been used for 30 years in Japan and South Korea. It is a drug focused on treating rheumatoid arthritis. Our company took the drug and repurposed it back in 2015 – where we ran a Phase IIa study in gout with it [2]. The drug showed safety and efficacy but at that time it did not continue down a clinical path with the drug, and we shelved the intellectual property.

When I came on board I looked into this drug – it has a history of being an anti-oxidant, antirheumatic, anti-inflammatory and some of the preclinical work in this drug in the USA goes back to the early-mid 2000s, although it’s never been available in North America. After looking at the data we have from this gout study and at the history of the drug, I felt that it could be a potential treatment for COVID.

Some of the arthritic drugs from some of the other companies are more on the intravenous level, whereas this drug can be taken in tablet form. This drug played a key role in the past in terms of being a strong anti-inflammatory, which could be of benefit to COVID patients, specifically those with inflammation. We went to the FDA in late summer 2020 and we were looking to do a Phase II study. However, they looked at our data and the work we have done with gout and our open investigational new drug application and they fast tracked us into conducting a Phase III study which we are currently involved in.

The safety has been good on the drug so far in this trial, which is planning to enrol up to about 1000 patients across up to 50 locations in the USA.

## Could you tell me more about the clinical trial & its primary goals & objectives?

The trial began as a Phase III, multicenter, randomized, double-blind, placebo-controlled, clinical study of bucillamine (two dosage levels) in patients with mild to moderate COVID-19. Patients were randomized 1:1:1 to receive 100 mg bucillamine three times a day (TID), 200 mg bucillamine TID or placebo TID for up to 14 days. The Drug and Safety Monitoring Board (DSMB) has selected 600 mg as the dose to go forward during a previous interim analysis. Patients since the 600 mg dose was chosen will be randomized 2:1 to the selected bucillamine dose or placebo. This is a double blinded randomized 1000 patient study.

The end points of the study are based on reducing hospitalizations and death for mild to moderate patients. Our goal is to show the safety of this drug as we reach some of these end points, and to demonstrate efficacy in these mild to moderate patients. We will not know the efficacy until the end, but safety has been good so far.

The drug potentially has capability on the antiviral and prophylactic side as studied at the University of California, San Francisco [3], where they showed that thiol drugs like bucillamine may help prevent the binding of the virus to the ACE2 receptors, but most of the focus has been on its anti-oxidant and anti-inflammatory capabilities. The goal here is to show the efficacy of our small-molecule drug. There are a number of different treatments like monoclonal antibodies, areas that are more on an intravenous level from some of the other companies. However, there is a great need to improve logistics, and increase the ease of use by giving somebody a small-molecule drug, where they could take it home and treat themselves so that they can prevent themselves from developing greater severity of the disease. Right now, there are only a handful of companies in the world that are looking in this area. For example, some major players such as Pfizer and Merck are looking at drugs on the antiviral side, such as molnupiravir, and those are scheduled to start trials or are in current trials. We are also in a Phase III trial ourselves, which puts us in a camp with the major players and our drug method of action is around anti-inflammatory and as an antioxidant. So there is an opportunity for the drug to stand alone, but it could also work in combination with another antiviral. Revive is the only company that has a drug in a Phase III trial for COVID, focused on the anti-inflammatory side.

## What are the unknowns associated with bucillamine? Are there any major known side effects related to bucillamine treatment?

As I mentioned bucillamine has been around for 30 years; it has a very good safety and it has been used in Japan and South Korea. It has not been made available in the USA, but it has a very good safety profile and so far, that has been exhibited in our Phase III study in the USA. The safety of this drug has been very strong and well documented so that gives us a strong standing in this trial, specifically with the regulatory agents like the DSMB and the FDA. As we carry forward down the trial, the safety profile really holds a lot of weight in terms of moving us down the process.

## Do you think there could be potential for bucillamine to also be applied in the treatment of other diseases?

Bucillamine has a history in rheumatoid arthritis – there has been preclinical work done with the drug a long time ago in different areas to help treat inflammation like reperfusion injury, areas like that. Obviously, we think that in gout it could have a strong role, because we did run a Phase IIa study with the drug in 2015. Bucillamine is a versatile drug so it could work with a number of other lung disorders potentially like cystic fibrosis and areas like that. In terms of infectious diseases it could be applied to, infectious diseases is a broad term, so when we do these trials, we have got to be very focused – the focus is on COVID at the moment, but the opportunity exists down the line to treat a number of different areas with this drug because it is quite versatile. It could have the potential to be applied to other infectious diseases that are associated with inflammation.
